# Dosimetric Comparison of Intensity-Modulated Radiotherapy, Volumetric Modulated Arc Therapy and Hybrid Three-Dimensional Conformal Radiotherapy/Intensity-Modulated Radiotherapy Techniques for Right Breast Cancer

**DOI:** 10.3390/jcm9123884

**Published:** 2020-11-29

**Authors:** Yi-Chi Liu, Hung-Ming Chang, Hsin-Hon Lin, Chia-Chun Lu, Lu-Han Lai

**Affiliations:** 1 Institute of Nuclear Engineering and Science, National Tsing Hua University, Hsinchu 30015 Taiwan; A21665@weigong.org.tw; 2 Department of Radiation Oncology, Wei Gong Memorial Hospital, Miaoli 35148, Taiwan; 3 Department of General Surgery, Wei Gong Memorial Hospital, Miaoli 35159, Taiwan; mikledoctor@weigong.org.tw; 4 Medical Physics Research Center, Institute for Radiological Research, Chang Gung University/Chang Gung Memorial Hospital, Taoyuan 33302, Taiwan; hh.lin@mx.nthu.edu.tw; 5 Department of Radiation Oncology, Chang Gung Memorial Hospital, Taoyuan 33305, Taiwan; 6 Department of Nuclear Medicine, Keelung Chang Gung Memorial Hospital, Keelung 20401, Taiwan; 7 Department of Biomedical Engineering and Environmental Sciences, National Tsing Hua University, Hsinchu 30013, Taiwan; s105012805@m105.nthu.edu.tw; 8 Department of Medical Imaging and Radiological Technology, Yuanpei University of Medical Technology, Hsinchu 30015, Taiwan

**Keywords:** right breast cancer, volumetric modulated arc therapy, intensity-modulated radiotherapy, hybrid 3D-CRT/IMRT

## Abstract

This study aimed to compare different types of right breast cancer radiotherapy planning techniques and to estimate the whole-body effective doses and the critical organ absorbed doses. The three planning techniques are intensity-modulated radiotherapy (IMRT), volumetric modulated arc therapy (VMAT; two methods) and hybrid 3D-CRT/IMRT (three-dimensional conformal radiotherapy/intensity-modulated radiotherapy). The VMAT technique includes two methods to deliver a dose: non-continuous partial arc and continuous partial arc. A thermoluminescent dosimeter (TLD) is placed in the RANDO phantom to estimate the organ absorbed dose. Each planning technique applies 50.4 Gy prescription dose and treats critical organs, including the lung and heart. Dose-volume histogram was used to show the planning target volume (V95%), homogeneity index (HI), conformity index (CI), and other optimized indices. The estimation of whole-body effective dose was based on the International Commission on Radiation Protection (ICRP) Publication 60 and 103. The results were as follows: Continuous partial arc and non-continuous partial arc showed the best CI and HI. The heart absorbed doses in the continuous partial arc and hybrid 3D-CRT/IMRT were 0.07 ± 0.01% and 0% (V5% and V10%, respectively). The mean dose of the heart was lowest in hybrid 3D-CRT/IMRT (1.47 Gy ± 0.02). The dose in the left contralateral lung (V5%) was lowest in continuous partial arc (0%). The right ipsilateral lung average dose and V20% are lowest in continuous partial arc. Hybrid 3D-CRT/IMRT has the lowest mean dose to contralateral breast (organs at risk). The whole-body effective doses for ICRP-60 and ICRP-103 were highest in continuous partial arc (2.01 Sv ± 0.23 and 2.89 Sv ± 0.15, respectively). In conclusion, the use of VMAT with continuous arc has a lower risk of radiation pneumonia, while hybrid 3D-CRT/IMRT attain lower secondary malignancy risk and cardiovascular complications.

## 1. Introduction

With the development of diagnostic medical imaging, more early-stage breast cancers could be detected. Breast-conserving surgery with radiotherapy is a method to treat early-stage breast cancer, and the patient’s mental health and quality of life is improved because radiotherapy provides effective promotion for treatment and survival [1,2,3]. The therapeutic techniques for breast cancer vary. Traditional 3D conformal radiotherapy (3D-CRT) uses the tangential fields method, in which it is difficult to achieve treatment target conformity and uniform dose distribution and leads to more irradiation around the target or normal tissue like the lung and heart and mores tissue damage and complications [4,5,6,7,8,9,10,11,12,13,14,15,16]. Intensity-modulated radiotherapy (IMRT) uses a multi-leaf collimator (MLC) and inverse treatment planning to modulate beam flux intensity to improve target conformity and lower irradiation dose to critical organs [17,18,19]. Volumetric modulated arc radiotherapy (VMAT) [20,21,22,23] is a technique that uses single or multi-arc rotating irradiation. During irradiation, position, speed, beam dose rate, and gantry rotation speed on MLC can be modulated to achieve higher target conformity and treatment efficiency [24].

In radiation therapy today, IMRT and VMAT are commonly used in treatment of cancers, Compared to traditional 2D and 3D CRT treatment techniques, however, IMRT and VMAT in which each beam is subdivided into multiple segments with differing MLC shapes require more monitor units (MU), where MU corresponds to the actual dose delivery to a patient. The increase will lead to the increase of low dose radiation to the rest of the body and increased risk of secondary radiation-induced malignancies [20,21,22,23,24]. To reduce lung irradiation dose, other techniques, such as non-continuous partial arc [25] and hybrid 3D-CRT/IMRT, are used. The dosimetry of patients with left breast cancer have been discussed in many literatures [26,27,28,29,30] to avoid unnecessary radiotherapy to the tissues of the heart. However, studies on the dosimetry of right breast radiotherapy were limited. Care should also be taken for the right breast radiotherapy as most hearts are located nearly in the center of the chest or close to the right side, such as cardiac hypertrophy or situs inversus. In this study, we aim to studying the dosimetry of right breast cancer by comparing different radiotherapy planning techniques. This study compared three types of radiotherapy, IMRT, VMAT and hybrid 3D-CRT/IMRT, in the treatment of early-stage right breast cancer. The result can be referenced as the choice of organ at risk (OAR) of optimized clinical treatment.

## 2. Experimental Section

### 2.1. Treatment Planning Selection

The VMAT technique includes two methods of delivering a dose: non-continuous partial arc and continuous partial arc. All treatment plans are executed for right breast cancer after breast-conserving surgery and designed by the same medical physicist, the difference in dose-volume histogram (DVH) of these plans are to be compared.

### 2.2. Mould Fabrication and Computed Tomography (CT) Simulation

The RANDO phantom lies on a vacuum bag; fix its position, and mark the metal wire on the left, right, and above the skin to be isocentric in the treatment plan. Use the Philips Medical System Brilliance Big Bore CT simulator with a scanning width of 0.5 cm. CT images were scanned and obtained from the mandible to umbilicus and transferred to a Philips Pinnacle3 version 9 RTP workstation.

### 2.3. Target Volume and Planning Organs-at-Risk Definition

The clinical target volume (CTV) of whole-breast irradiation includes the whole breast tissue, lymph nodes, front border of 0.5 cm beneath the skin, and area of tumor metastasis. The planning target volume (PTV) is the CTV area extending to 0.7 cm except the front border and the area considering the distance error during breath or positioning. The planning organ-at-risk volume includes patient’s ipsilateral lung, contralateral lung, heart, spinal cord, and thyroid.

### 2.4. Design of the Treatment Plan

**Figure 1:**
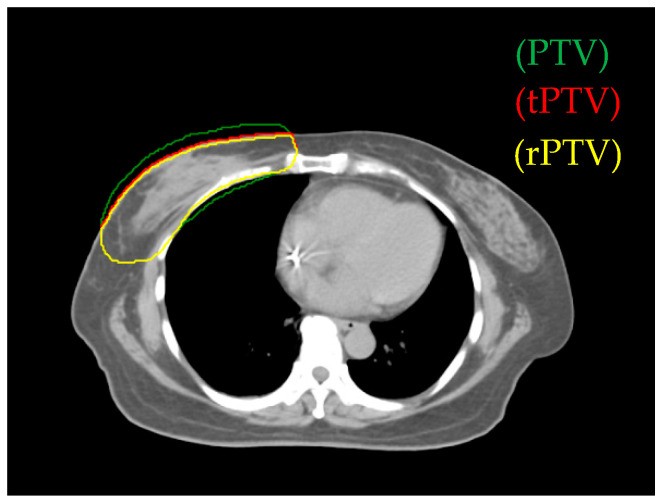
The green area is the planning target volume (PTV), the red area is the true PTV (tPTV), and yellow area is the reduced PTV (rPTV).

The software used for treatment planning was the Pinnacle3 version 9 RTP. We defined true PTV (tPTV) as the effective PTV margin delineated by the physician after excluding the error region (the part of air and lung). To decrease the surface for achieving greater skin sparing, 0.3 cm retraction of tPTV at the upper border was applied and defined as reduced PTV (rPTV) (see Figure 1). The planning technique extended rPTV to 0.5 cm and added 0.5 cm width in 90% prescription dose constraint circle and 1cm gap as addition area. In all plans, the prescription dose was 50.4 Gy in 28 fractions to the right breast PTV (Table 1). The Elekta Synergy medical linear accelerator was used, the photon energy was 6 MV.

### 2.5. IMRT Treatment Planning

**Figure 2:**
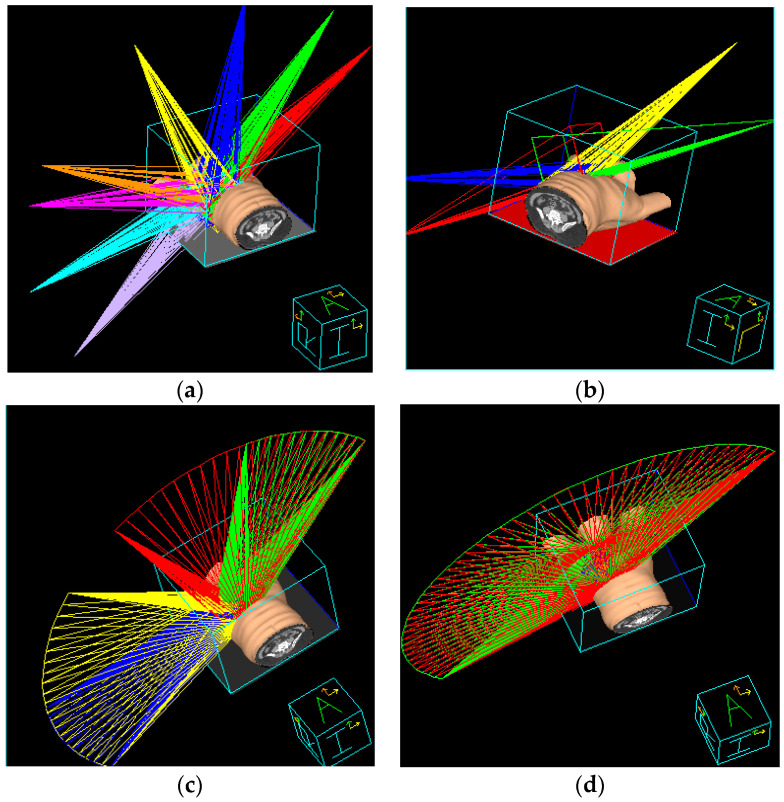
Treatment planning using (**a**) intensity-modulated radiotherapy (IMRT), (**b**) hybrid 3D conformal radiotherapy (3D-CRT)/IMRT, (**c**) non-continuous partial arc, and (**d**) continuous partial arc.

Two tangential angles were designed according to target shape and maximum sparing of lung volume. Initially, the left and right tangential angles were 235° and 55°, respectively. An irradiation field is set with an increment in tangential angles, and each separated by 20°–30°. There is a total of eight irradiation fields (Figure 2a). The IMRT used the direct machine parameter optimization algorithm.

### 2.6. Hybrid 3D-CRT/IMRT Treatment Planning

This planning was combined with two respective irradiation fields of 3DCRT and IMRT. 3DCRT irradiation field was set with two tangential angles. In the Beam’s Eye View software, the MLC, lead blocks, and collimator angles were regulated manually to obtain an appropriate irradiation field. The optimized lung shield was also considered to decrease radiation dose. Given a 70% prescription dose (Figure 2b), 180 cGy was irradiated, in a total of 28 fractions to obtain an adequate dose for 95% PTV. After 3DCRT is completed, with the rest of the 30% at prescription dose with the same irradiation method, two IMRT fields were added with increment of tangential angles; each was separated at 20°.

### 2.7. Non-Continuous Partial Arc Treatment Planning

To reduce unwanted irradiation on the lung, the method uses partial rotation treatment. The right tangential angle was counter clockwise at 55°–335° and 275°–235°, and the left tangential angle was clockwise at 235°–315° and 15°–55°, with a total of 4 arc rotating irradiation fields (Figure 2c). The plan uses the SmartARC algorithm.

### 2.8. Continuous Partial Arc Treatment Planning

The first irradiation field was rotated counter clockwise, starting from the right to the left tangential angle. The second irradiation field was in counter-rotating direction as that of the first field. The treatment plan had two half-arc continuous irradiation fields (Figure 2d).

### 2.9. Organ Absorbed Dose and Whole-Body Effective Dose Measurement

**Figure 3:**
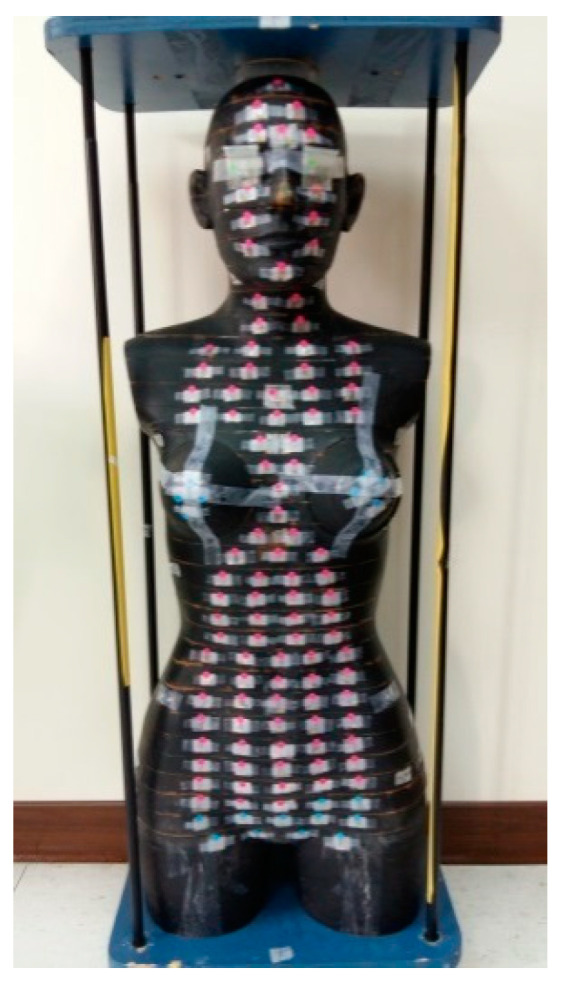
RANDO phantom.

The present study used arc treatment planning. Low-dose scattering was possibly induced by multi-angle incident. Organs around target may accept the irradiation dose. RANDO phantom (tissue density, 0.985 g/cm^3^; effective atomic number, 7.3) with thermoluminescent dosimeter (TLD) (TLD-100H) (100 mg/cm^2^) was used. With the medical linear accelerator in standard status [31] (SAD 100 cm with irradiation field 10 × 10 cm, 1 cGy = 1 MU), ±5% TLD was selected; a set consisted of three TLDs. TLD was placed in main body organs (Figure 3) and in radiation-sensitive organs recommended by the International Commission on Radiation Protection (ICRP) [32,33], and the absorbed dose was converted to effective dose using Equations (1) and (2).

(1) $$H_{T}=D_{T}\times W_{R}$$

(2) $$E=\sum_{T}W_{T}H_{T}$$

*H_T_* is the tissue equivalent dose, and the unit is Sv. *D_T_* is the respective organ absorbed dose, and the unit is Gy. *W_R_* is the radiation weighting factor (*W_R_*) based on ICRP (*W_R_* for photon is 1). *W_T_* is the tissue *W_R_*. Equation (1) was applied to convert the absorbed dose (Gy) to equivalent dose (Sv), and Equation (2) was applied to convert the equivalent dose (Sv) to effective dose (Sv). The thin-film TLD GR-200F (5 mg/cm^2^) was placed on the RANDO phantom surface to measure absorbed dose in the skin.

The effective dose was obtained by summing the multiplications of each tissue equivalent dose by each tissue weighting factor defined in ICRP-60 and ICRP-103.

### 2.10. Dosimetry Parameters Comparison among Treatment Plans

Four treatment plans use DVH to compare dosimetry parameters on target volume, including mean dose, maximum dose, minimum dose, V110%, V107%, V105%, V100%, and V95%. The conformity index (CI) [34] was used to indicate the conformity of the treatment plan, as shown in Equation (3).

(3) $$\mathrm{CI}=\frac{TV_{RI}\times TV_{RI}}{TV\times V_{RI}}$$

*TV_RI_*: PTV in 95% prescription dose;
*TV*: PTV;
*V_RI_*: whole volume in 95% prescription dose;
CI ranged from 0 to 1, and the plan is more conformal as CI gets closer to 1.

The homogeneity index (HI) indicated the dose homogeneity in the target volume and was mainly used to analyze the volume ratio for high dose V105% (called HI_1_) in the tumor, as shown in Equation (4) [27]. As HI_1_ gets closer to 1, the dose difference in target volume becomes smaller, and dose distribution becomes more homogeneous.

(4) $$\mathrm{HI}=\left(\frac{V_{PTV,95\%\;dose}}{V_{PTV}}\right)\times \left(\frac{1-V_{PTV,105\%\;dose}}{V_{PTV}}\right)$$

*V_PTV_*_,95%_*_dose_*: Volume reaching 95% prescription dose in target volume;
*V_PTV_*_,105% *dose*_: Volume reaching 105% prescription dose in target volume;
*V_PTV_*: PTV.

Another HI (called HI_2_) [35] was mainly used to analyze the ratio difference between D_2_ (absorbed dose in 2% PTV) to prescription dose and D_98_ (absorbed dose in 98% PTV) to prescription dose, as shown in Equation (5). As HI_2_ gets closer to 0, the dose difference in target volume becomes smaller, and dose distribution becomes more homogeneous.

(5) $$\mathrm{HI}=\frac{D_{2\%}\times D_{98\%}}{D_{P}}\times 100\%$$

*D*_2%_: Maximum absorbed dose in 2% PTV;
*D*_98%_: Minimum absorbed dose in 98% PTV;
*D_P_*: Prescription dose.

In the heart, ipsilateral lung, contralateral lung, and contralateral breast, DVH was used to compare respective parameters, including mean dose, maximum dose, minimum dose, V5Gy (%), V10Gy (%), V20Gy (%), V30Gy (%), V40Gy (%), and V50.04Gy (%). In the planning, it is also used to estimate respective optimized parameters, such as delivery time and MU.

## 3. Results

### 3.1. Comparison of Reduced PTV (rPTV) in Four Techniques on Dosimetry

PTC Coverage rate and CI based on V95% are the main criteria for treatment planning (Table 2). In each planning technique, the mean r-PTV doses with standard deviation were 52.05 Gy ± 0.09, 51.61 Gy ± 1.33, 51.82 Gy ± 1.52, and 51.81 Gy ± 1.45 in continuous partial arc, non-continuous partial arc, hybrid 3D-CRT/IMRT, and IMRT, respectively. V95% for continuous partial arc, non-continuous partial arc, hybrid 3D-CRT/IMRT, and IMRT were 97.42% ± 0.07, 97.41% ± 0.90, 95.77% ± 1.18, and 96.62% ± 2.01, respectively. Continuous partial arc had the best coverage rate in planning target area. Hybrid 3D-CRT/IMRT had slightly less but still reached 95%. CIs were 0.74 ± 0.01, 0.74 ± 0.01, 0.68 ± 0.03, and 0.64 ± 0.05, respectively. CIs in continuous partial arc and non-continuous partial arc were better than that in IMRT (from 0.64 to 0.74). In non-continuous partial arc, HI_1_ was 0.89 ± 0.11, and HI_2_ was 12.79 ± 0.03; both are better than those in the other three planning techniques.

### 3.2. Normal Tissue Comparison on Dosimetry

#### 3.2.1. Heart

**Figure 4:**
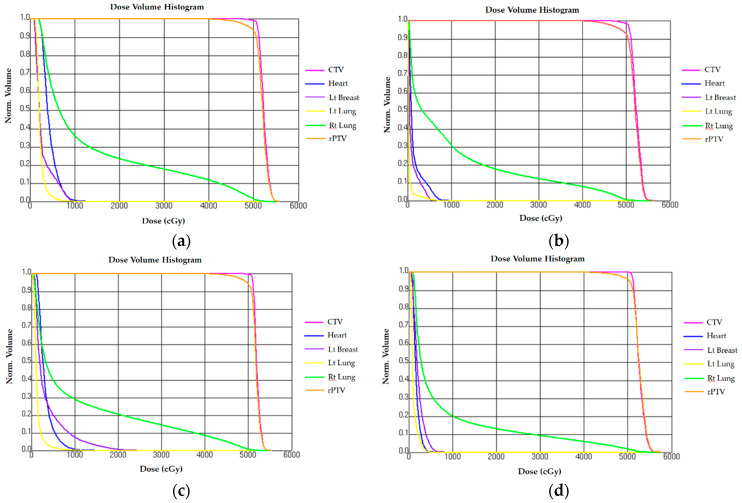
Dose-volume histogram of (**a**) IMRT, (**b**) hybrid 3D-CRT/IMRT, (**c**) non-continuous partial arc, and (**d**) continuous partial arc. CTV, clinical target volume; PTV, planning target volume.

The constraint for heart dose was V17.64Gy (%) <35%. The study showed that mean heart doses (Gy) for continuous partial arc, non-continuous partial arc, hybrid 3D-CRT/IMRT, and IMRT were 1.73 Gy ± 0.07, 3.15 Gy ± 0.03, 1.47 Gy ± 0.02, and 4.35 Gy ± 0.01, respectively. V10 Gy (%) were 0.7% ± 0.03 in the non-continuous partial arc, 0.0048% ± 0.01 in IMRT, and 0% in other planning techniques. The mean heart dose in continuous partial arc and hybrid 3D-CRT/IMRT was <2 Gy, while that in non-continuous partial arc and IMRT was >3 Gy. The lowest mean heart dose was measured for the hybrid 3D-CRT/IMRT (Table 3 and Figure 4).

#### 3.2.2. Ipsilateral Lung

The constraint for right lung dose was V22.68Gy (%) <35%. The study showed that the mean dose of ipsilateral lung (Gy) for continuous partial arc, non-continuous partial arc, hybrid 3D-CRT/IMRT, and IMRT were 8.32 Gy ± 1.6, 10.71 Gy ± 0.09, 10.14 Gy ± 1.47, and 12.75 Gy ± 1.23, respectively. The mean dose of right lung was lowest in continuous partial arc and highest in IMRT (Table 3 and Figure 4).

#### 3.2.3. Contralateral Lung

The constraint for left lung dose was V17.64Gy (%) <20%. The study showed that the mean dose of contralateral lung (Gy) for continuous partial arc, non-continuous partial arc, hybrid 3D-CRT/IMRT, and IMRT were 1.19 ± 0.56, 1.31 ± 0.71, 0.42 ± 0.23, and 2.25 ± 0.05, respectively. The mean dose of left lung was lowest in hybrid 3D-CRT/IMRT and highest in IMRT. (Table 3 and Figure 4).

#### 3.2.4. Contralateral Breast

The mean dose of the left breast (Gy) was highest in non-continuous partial arc (3.51 ± 1.25) but lowest in hybrid 3D-CRT/IMRT (0.98 ± 0.15). (Table 3 and Figure 4).

#### 3.2.5. Comparison of Planning Parameter Optimization

In all four planning techniques, delivery time in continuous partial arc is the shortest (approximately 234 ± 15 s) and that in IMRT is the longest (approximately 300 ± 35 s). Shorter treatment time can reduce the risk of patient motion and discomfortable. MU was highest in continuous partial arc (687 ± 15) and lowest in hybrid 3D-CRT/IMRT (319.1 ± 19) (Table 4). This large MU value was caused by the high modulation of MLCs and may lead to increase the risk of secondary cancer.

#### 3.2.6. Comparison between Organ Absorbed Dose and Whole-Body Effective Dose

The doses in the eye lens in continuous partial arc, non-continuous partial arc, hybrid 3D-CRT/IMRT, and IMRT were 4.7 Gy ± 0.03, 0.38 Gy ± 0.02, 0.46 Gy ± 0.02, and 4.32 Gy ± 0.04, respectively (Table 5). The lens dose was highest in continuous partial arc. The skin dose was highest in IMRT. The effective dose of treatment planning of continuous partial arc is the highest among the four treatment plans (Table 5). Hence, attention should be paid to the higher scattering dose that contributes to the eye lens and the effective dose, when the continuous partial arc is used.

## 4. Discussion

As hybrid 3D-CRT/IMRT treatment planning is applied on early-stage breast-conserving surgery, hybrid 3D-CRT/IMRT and IMRT achieved better CI, lower high-dose area, and increase target area homogeneity [36]. Moreover, 3DCRT and IMRT have worse dose homogeneity, and hybrid 3D-CRT/IMRT had better [37]. The experiment result is consistent with those in literature.

Pericarditis may develop as the heart is irradiated, but it rarely progresses in a short duration [38]. In the treatment of breast cancer, some patients have a long-term follow-up. It was found that, with a mean heart dose of 4.9 Gy, the risk of developing cardiovascular disease increases to 7.4% for each 1 Gy additional dose [39]. The study result shows that, when V30Gy (%) is >46%, the risk of pericarditis is 73% [40]. In continuous partial arc, the mean heart dose is 4.6 Gy ± 1.7, V5Gy (%) is 26.1% ± 15.1, and V10Gy (%) is 6.9% ± 4.9 [33]. In 3DCRT, the mean heart dose is 4.39 Gy ± 2.24, V5Gy (%) is 10.30% ± 6.27, and V10Gy (%) is 7.50% ± 5.43 [22]. In the non-continuous partial arc, the mean heart dose is 7.61 Gy ± 1.38, V5Gy (%) is 59.73% ± 15.87, and V10Gy (%) is 24.39% ± 6.82. The planning heart mean dose in our study is almost <4 Gy, except for that in IMRT. Continuous partial arc and hybrid 3D-CRT/IMRT had lower mean heart dose, especially hybrid 3D-CRT/IMRT.

**Figure 5:**
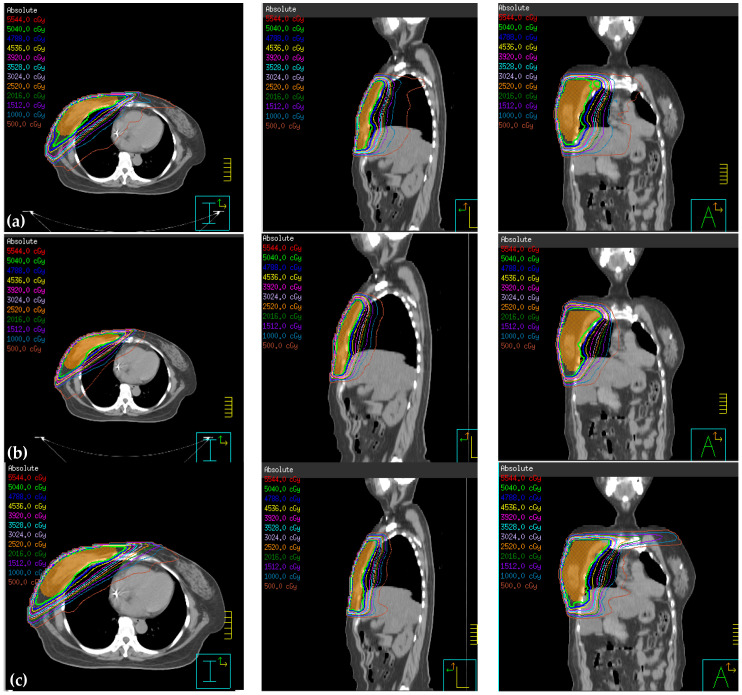

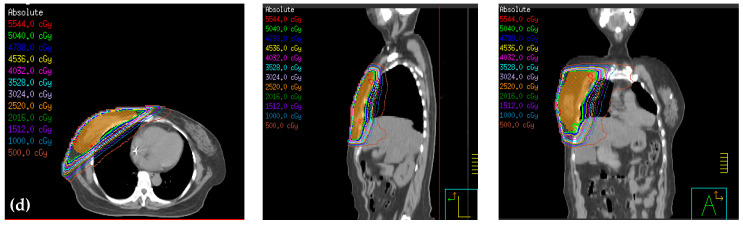
Dose distribution of (**a**) IMRT, (**b**) hybrid 3D-CRT/IMRT, (**c**) non-continuous partial arc, and (**d**) continuous partial arc.

In the treatment of breast cancer, radiation pneumonitis (RP), in which the lung tissue in the irradiation field is damaged, may cause respiratory injury [41]. When the lung is irradiated V20Gy (%) of 20%, 21–25%, 26–30%, and >31%, the risk of developing RP in half year is 8.7%, <18.3%, 51%, and 85%, respectively [40]. When the lung is irradiated V20Gy (%) <22%, 22–30%, 31–40%, and >40%, the risk of developing RP in 2 years is 0%, <7%, 13%, and 36%, respectively [42]. In continuous partial arc, the mean ipsilateral lung dose is 10.10 Gy ± 2.5, V5Gy (%) is 50.3% ± 13.3, V10Gy (%) is 29.9% ± 8.0, and V20Gy (%) is 16.4%±4.8 [43]. In non-continuous partial arc, the mean ipsilateral lung dose is 8.22 Gy ± 0.57, V5Gy (%) is 40.46% ± 3.81, V10Gy (%) is 23.32% ± 2.07, and V20Gy (%) is 12.71% ± 2.23 [25]. Studies showed that, when the mean lung dose is <10 Gy, 10–20 Gy, 20–30 Gy, and >30 Gy, the risk of developing RP is 10%, 16%, 27%, and 44%, respectively [42,44]. In this study, the ipsilateral lung dose of treatment planning of continuous partial arc is the lowest among the four treatment plans, while IMRT is the largest. V20% in continuous partial arc is also the lowest. Our results are consistent with that of the above-mentioned studies and reports lower ipsilateral lung dose when continuous partial arc was applied. The main reason of the low dose lies in the higher conformity provided from dual arc for continuous partial arc. This technique can enhance dose modulation in the irradiation field and beam usage rate. Staring from the tangential angle, the beam irradiated along the chest wall, the dose weight limit for the lung can be achieved when designing the plan, and dose irradiation on the lung is reduced. On the other hands, the angle design for IMRT can be considered with target area shape. The close area can also be irradiated if the target area is extremely close to the lung. Although there is intensity modulation in the irradiation field, it forms mainly with MLC. Leaves in the MLC induce dose scattering and radiation leakage and may increase neighboring organ dose (Figure 5).

According to the literature [45], the mean dose in the contralateral breast is 2.82 Gy. Whether the second breast cancer is induced should consider many factors. The risk of cancer recurrence is approximately 220–310 per million, and it may take 15 years to develop. hybrid 3D-CRT/IMRT has the lowest mean dose to contralateral breast, helping reduce the risk of secondary breast cancers.

In the planning techniques, organs absorbed dose and whole-body effective dose in both continuous partial arc and IMRT are highest in the lens and skin. Because the continuous partial arc is a dynamic arc treatment, continuous rotation easily induces large-area low-dose scattering and applies scattering dose to neighboring organs. In IMRT, an arc effect is also formed due to the small angle among beams. It has a similar result to that in continuous partial arc. Due to additional calculation of main organs (brain and salivary gland) and adjustment of tissue W_R_, all effective doses in ICRP-103 are higher than those in ICRP-60.

## 5. Conclusions

This study compared IMRT, hybrid 3D-CRT/IMRT, VMAT with non-continuous partial arc, and VMAT with continuous partial arc to treat right breast cancer, with the aim of determining which techniques provided the best target coverage while minimizing doses to the OARs. Of the four techniques investigated, the results indicate VMAT attain the best target coverage, hybrid 3D-CRT/IMRT has the lowest heart mean dose, while VMAT with continuous partial arc can achieve the lowest mean dose in lung area (left contralateral lung and the right ipsilateral lung). Therefore, the use of VMAT with continuous arc has a lower risk of radiation pneumonia, while hybrid 3D-CRT/IMRT attain lower secondary malignancy risk and cardiovascular complications. Both techniques are a good choice depending on the trade-off of more consideration of protections on the lung or heart.

## Figures and Tables

**Table 1:** Target dose and dose-volume constraint of the organs.

Target	Dose (cGy)		Volume (%)
PTV	Max dose	<5544	
	Max DVH	4788	100%
Heart	Max dose	<5292	
	Max DVH	1765	<35%
Ipsilateral lung	Max dose	<5544	
	Max DVH	2000	<25%
Contralateral lung	Max dose	<5292	
	Max DVH	1764	<20%

PTV: Planning target volume; DVH: Dose-volume histogram.

**Table 2:** Comparison of PTV dose-volume histogram (DVH) parameters in 4 different treatment plans.

Structures	Continuous Partial Arc	Non-Continuous Partial Arc	Hybrid 3D-CRT/IMRT	IMRT
Mean dose (Gy)	52.05 ± 0.09	51.61 ± 1.33	51.82 ± 1.52	51.81 ± 1.45
Maximum dose (Gy)	55.88 ± 0.16	55.07 ± 0.08	56.15 ± 0.07	55.52 ± 0.08
minimal dose (Gy)	25.60 ± 0.16	23.69 ± 1.23	21.67 ± 0.15	23.00 ± 0.04
V100 (%)	97.42 ± 0.09	92.39 ± 0.23	87.61 ± 1.23	89.12 ± 1.43
V95 (%)	97.42 ± 0.07	97.41 ± 0.9	95.77 ± 1.18	96.62 ± 2.01
V105 (%)	18.59 ± 0.14	18.81 ± 0.11	15.31 ± 1.01	14.39 ± 1.54
V107 (%)	2.05 ± 0.06	0.78 ± 0.04	2.44 ± 0.10	2.38 ± 0.07
V110 (%)	0.10 ± 0.11	0.00 ± 0.00	0.02 ± 0.01	0.01 ± 0.01
D2 (Gy)	53.93 ± 1.45	53.60 ± 2.73	54.25 ± 1.61	54.25 ± 1.48
D98 (Gy)	47.21 ± 0.98	47.15 ± 1.44	45.67 ± 1.77	46.4 ± 1.22
CI	0.74 ± 0.01	0.74 ± 0.01	0.68 ± 0.03	0.64 ± 0.05
HI_1_	0.79 ± 0.02	0.89 ± 0.11	0.71 ± 0.21	0.78 ± 0.11
HI_2_	13.33 ± 0.01	12.79 ± 0.03	17.02 ± 0.03	15.57 ± 0.04

Vx (%): x % of the prescribed dose volume; Dx (Gy): a volume received greater than x Gy; CI: conformity index; HI: Homogeneity Index.

**Table 3:** Comparison of normal tissue DVH parameters in four different treatment plans.

Structures	Continuous Partial Arc	Non-Continuous Partial Arc	Hybrid 3D-CRT/IMRT	IMRT
**Heart**				
Mean dose (Gy)	1.73 ± 0.07	3.15 ± 0.03	1.47 ± 0.02	4.35 ± 0.01
minimal dose (Gy)	0.71 ± 0.04	1.04 ± 0.04	0.30 ± 0.01	1.93 ± 0.03
Maximum dose (Gy)	5.82 ± 0.02	1.47 ± 0.07	9.52 ± 0.08	12.38 ± 0.06
V5Gy (%)	0.07 ± 0.01	0.12 ± 0.01	0.07 ± 0.01	0.27 ± 0.03
V10Gy (%)	0.00 ± 0.00	0.70 ± 0.03	0.00 ± 0.00	0.005 ± 0.01
V50.40Gy (%)	0.00 ± 0.00	0.00 ± 0.00	0.00 ± 0.00	0.00 ± 0.00
**Right lung (ipsilateral)**				
Mean dose (Gy)	8.32 ± 1.61	10.71 ± 0.09	10.14 ± 1.47	12.76 ± 1.23
minimal dose (Gy)	0.80 ± 0.04	0.50 ± 0.03	0.27 ± 0.01	1.65 ± 0.01
Maximum dose (Gy)	54.06 ± 1.08	52.32 ± 0.06	51.88 ± 0.09	52.37 ± 0.05
V5Gy (%)	32.56 ± 0.91	39.69 ± 0.02	45.12 ± 0.77	60.82 ± 0.32
V10Gy (%)	20.02 ± 0.13	8.96 ± 0.10	30.97 ± 0.58	35.80 ± 0.18
V20Gy (%)	12.96 ± 0.34	20.43 ± 1.00	17.55 ± 1.69	23.33 ± 1.91
V30Gy (%)	9.16 ± 0.45	14.30 ± 0.09	12.09 ± 1.01	17.54 ± 0.09
V40Gy (%)	5.80 ± 0.69	8.38 ± 0.45	7.69 ± 0.33	11.60 ± 0.17
V50.40Gy (%)	1.01 ± 0.09	0.24 ± 0.01	0.19 ± 0.04	0.79 ± 0.01
**Left lung (contralateral)**				
Mean dose (Gy)	1.19 ± 0.56	1.31 ± 0.71	0.42 ± 0.23	2.25 ± 0.05
minimal dose (Gy)	0.43 ± 0.05	0.21 ± 0.07	1.02 ± 0.02	0.83 ± 0.01
Maximum dose (Gy)	5.27 ± 0.03	11.65 ± 0.17	6.89 ± 0.09	10.09 ± 0.12
V5Gy (%)	0.00 ± 0.00	1.79 ± 0.04	0.67 ± 0.05	2.57 ± 0.03
V10Gy (%)	0.00 ± 0.00	0.03 ± 0.01	0.00 ± 0.00	0.00 ± 0.00
V50.40Gy (%)	0.00 ± 0.00	0.00 ± 0.00	0.00 ± 0.00	0.00 ± 0.00
**Left breast**				
Mean dose (Gy)	2.26 ± 0.13	3.51 ± 1.25	0.98 ± 0.15	2.78 ± 0.17
minimal dose (Gy)	0.41 ± 0.08	0.19 ± 0.09	0.22 ± 0.09	0.36 ± 0.04
Maximum dose (Gy)	8.39 ± 0.32	24.49 ± 1.33	6.60 ± 1.12	9.60 ± 1.66
V5Gy (%)	3.44 ± 0.13	20.13 ± 0.07	1.25 ± 0.08	14.90 ± 0.09
V10Gy (%)	0.00 ± 0.00	7.53 ± 0.09	0.00 ± 0.00	0.00 ± 0.00
V50.40Gy (%)	0.00 ± 0.00	0.00 ± 0.00	0.00 ± 0.00	0.00 ± 0.00

VxGy (%): Vx means the percent of volume receiving x or more Gy. Note: V20Gy (%), V30Gy (%), and V40Gy (%) of normal tissue were not tabulated once these parameters for four different treatment plans were all zero.

**Table 4:** Comparison of parameters for 4 different treatment techniques.

Machine Parameters	Continuous Partial Arc	Non-Continuous Partial Arc	Hybrid 3D-CRT/IMRT	IMRT
Calculation mode	SmartArc	SmartArc	DMPO	DMPO
Maximum number of segments	40	40	25	25
Gantry angle	Beam 1: 55 ± 10°–235 ± 10° Beam 2: 235 ± 10°–55 ± 10°	Beam 1: 55 ± 10°–335 ± 10° Beam 2: 275 ± 10°–235 ± 10° Beam 3: 235 ± 10°–315 ± 10° Beam 4: 15 ± 10°–55 ± 10°	Beam 1: 3D–235 ± 10° Beam 2: 3D–55 ± 10° Beam 3: IMRT–250 ± 10° Beam 4: IMRT–35°	Beam 1: 56° Beam 2: 37° Beam 3: 7° Beam 4: 333° Beam 5: 284° Beam 6: 257° Beam 7: 229° Beam 8: 295°
Collimator angle	10°	10°	3D: 10° 3D: 350°	×
Wedge	×	×	×	×
Delivery time (s)	234 ± 15	146 ± 11	245 ± 20	300 ± 35
Delivery MU	687 ± 15	519.4 ± 17	319.1 ± 19	427.1 ± 25

×: It is not applicable in this technique.

**Table 5:** Summary of absorbed dose and effective dose in 4 different treatment plans.

Dose	Continuous Partial Arc	Non-Continuous Partial Arc	Hybrid 3D-CRT/IMRT	IMRT
Organ dose (Gy)				
Lens	4.7 ± 0.03	0.38 ± 0.01	0.46 ± 0.02	4.32 ± 0.04
Skin	2.34 ± 0.13	0.8 ± 0.09	1.65 ± 0.05	2.41 ± 0.01
Effective dose (Sv)				
ICRP-60	2.01 ± 0.23	0.72 ± 0.08	0.9 ± 0.03	1.88 ± 1.1
ICRP-103	2.89 ± 0.15	1.25 ± 0.1	1.48 ± 0.18	2.71 ± 0.6

ICRP-X: International Commission on Radiological Protection Publication X.
